# The ECRG4 cleavage product augurin binds the endotoxin receptor and influences the innate immune response during otitis media

**DOI:** 10.3389/fgene.2022.932555

**Published:** 2022-08-26

**Authors:** Arwa Kurabi, Dong Gu Hur, Kwang Pak, Madeline Gibson, Nicholas J. G. Webster, Andrew Baird, Brian P. Eliceiri, Allen F. Ryan

**Affiliations:** ^1^ Department of Otolaryngology, University of California, San Diego, La Jolla, CA, United States; ^2^ Department of Otorhinolaryngology, Gyeongsang National University Changwon Hospital, Changwon, South Korea; ^3^ Department of Medicine, University of California, San Diego, La Jolla, CA, United States; ^4^ San Diego Veterans Administration Healthcare System, San Diego, CA, United States; ^5^ Department of Surgery, University of California, San Diego, La Jolla, CA, United States

**Keywords:** bacterial middle ear infection, NTHi, growth suppression, ECRG4, augurin

## Abstract

Otitis media (OM), the most common disease of childhood, is typically characterized by bacterial infection of the middle ear (ME). Prominent features of OM include hyperplasia of the ME mucosa, which transforms from a monolayer of simple squamous epithelium with minimal stroma into a full-thickness respiratory epithelium in 2–3 days after infection. Analysis of the murine ME transcriptome during OM showed down-regulation of the tumor suppressor gene *Ecrg4* that was temporally related to mucosal hyperplasia and identified stromal cells as the primary ECRG4 source. The reduction in *Ecrg4* gene expression coincided with the cleavage of ECRG4 protein to release an extracellular fragment, augurin. The duration of mucosal hyperplasia during OM was greater in *Ecrg4* ^*−/−*^ mice, the number of infiltrating macrophages was enhanced, and ME infection cleared more rapidly. ECRG4-null macrophages showed increased bacterial phagocytosis. Co-immunoprecipitation identified an association of augurin with TLR4, CD14 and MD2, the components of the lipopolysaccharide (LPS) receptor. The results suggest that full-length ECRG4 is a sentinel molecule that potentially inhibits growth of the ME stroma. Processing of ECRG4 protein during inflammation, coupled with a decline in *Ecrg4* gene expression, also influences the behavior of cells that do not express the gene, limiting the production of growth factors by epithelial and endothelial cells, as well as the activity of macrophages.

## Introduction

Otitis media (OM), the most common childhood disease, affects almost all children (Casselbrandt et al., 1993). While most OM are self-limiting, resolving within a few days even without treatment, 10–15% of children experience recurrent or chronic disease (Teele et al., 1989; Kaur et al., 2017). Ear infections cause more physician visits, surgeries, and antibiotic prescriptions than any other pediatric condition (Rovers, 2008). OM, with incidence peaking between the ages of 6 months and 2 years but remaining high until 6–7 years (Pichichero, 2016), also leads to hearing loss. This coincides with critical periods of speech and language gain, development of the central auditory system, and learning. Thus, chronic/recurrent OM has been found to be associated with speech (Friel-Patti and Finitzo, 1993) and language delay (Klausen et al., 2000), as well as learning deficiencies (Williams and Jacobs, 2009) and central auditory processing disorders (Klein et al., 1988). OM results in substantial care costs; e.g., in the US, estimated direct costs are at approximately $5 billion/year (Teele et al., 1989; Rosenfeld and Bluestone, 1993; Tong et al., 2018). However, globally, OM is a much more serious problem in developing countries and where access to healthcare is limited. Undertreated OM can progress to labyrinthitis or even meningitis. The World Health Organization (WHO) estimates that half of all disabling hearing loss in the world is due to undertreated OM, plus an estimated 30,000 annual childhood deaths (WHO, 2004; Leach et al., 2020; WHO, 2020).

OM is multifactorial, with contributing etiologies that include Eustachian tube dysfunction, prior upper respiratory infection by viruses, genetic factors, and environment (Massa et al., 2021). However, these etiologies typically lead to the final common pathway of bacterial infection, primarily by non-typeable *Haemophilus influenzae* (NTHi), *Streptococcus pneumoniae* (SPN), and/or *Moraxella catarrhalis*, with some viral co-infection (Ruohola et al., 2006; Schilder et al., 2016)*.* Streptococcal vaccines have reduced ME infections by covered SPN strains, but OM is an opportunistic infection, and ME infection by other bacteria and by uncovered SPN strains has increased (Casey et al., 2010). As a result, OM incidence has declined modestly due to vaccination (Vojtek et al., 2017).

Bacterial infection causes dramatic pathological changes to the ME. This includes extensive hyperplasia of the ME mucosa. The epithelium lining the ME cavity transforms from a monolayer of simple squamous epithelium into a pseudostratified, columnar respiratory epithelium with secretory, goblet and ciliated cells within 1–2 days after infection. A thin stromal similarly grows into a thickened, well-organized, and vascularized subepithelial layer. Altogether, the ME mucosa can rapidly increase in thickness by 10–20-fold (Lim, 1979).

For most children, the resolution of OM occurs in a few days, even without antibiotic treatment (Teele et al., 1989; Kaur et al., 2017). This is too soon for adaptive immunity to be responsible for clearance of ME infection, indicating that innate immunity is the normal means of OM resolution. Supporting this concept, association studies have linked alleles of a number of innate immune genes to OM (Wiertsema et al., 2006; Emonts et al., 2007; Ilia et al., 2014; Nokso-Koivisto et al., 2014; Toivonen et al., 2017), while animal studies have found that deletion of these and other innate immune receptors delays OM resolution (Hernandez et al., 2008.; Leichtle et al., 2009, 2012; Kurabi et al., 2015). Innate immune activation also rapidly induces ME inflammation, including the expression of pro-inflammatory cytokines (Hernandez et al., 2015).

While innate immunity in OM has been extensively studied, the pathways that regulate mucosal hyperplasia remain incompletely understood. We previously assessed the potential role of growth factors in hyperplasia and identified heparin-binding epithelial growth factor (HB-EGF) as strongly up-regulated during ME mucosal growth (Hernandez et al., 2015) and more capable of stimulating the proliferation of mucosal explants in culture than other epithelial growth factors expressed during OM (Suzukawa et al., 2014). In addition, inhibition of HB-EGF reduced mucosal hyperplasia induced by polyI:C administration in the ME (Sakamoto et al., 2022). However, another potential mechanism of hyperplasia promotion is down-regulation of tissue growth suppression (e.g., Sun and Yang, 2010). The purpose of this study was to assess the expression and role of negative regulators of tissue growth during OM.

## Materials and methods

### Animals

ECRG4 knockout (KO) mice were purchased from the Mutant Mouse Regional Resource Center supported by the National Institutes of Health and distributed by the University of California, Davis. Exon 1 of the *Ecrg4* gene (RIKEN cDNA 1500015O10) was targeted by homologous recombination, generated on a B6/129S5 mixed background. The ECRG4 KO mice were then backcrossed to C57/BL6 mice (Jackson Laboratory) for five generations to generate homozygous ECRG4 KO mice on the C57/BL6 background. The KO were confirmed by genotyping (Transnetyx) and qPCR. Age-matched C57BL/6 (WT) mice were used for KO phenotype evaluation, gene arrays, and single-cell RNA-Seq (scRNA-Seq). Gene array studies were also performed on WT C57BL/6/WB F1 hybrids. All experiments were performed according to the National Institutes of Health guidelines and approved by the VA San Diego Medical Center IACUC.

### OM generation

MEs were surgically exposed using a ventral approach; a small hole was created in the bone of the ME bullae, and 5 × 10^3^ non-typeable *Haemophilus influenzae* (NTHi) strain 3655 in 5 μL PBS was injected into each ear. The ears were inspected after NTHi inoculation for TM perforations, in which case they were rejected. Otherwise, all animals were included. Animals were sacrificed, and MEs were evaluated at 0 h (0 h, uninfected), 6 h, 1 day (1d), 2d, 3d, 5d, 7d, or 10d. Animals were assigned to groups at random. Each time point group has at least six animals from each strain (ECRG4^−/−^; WT). At each time point, the ME contents were cultured for the presence of viable NTHi and evaluated histologically for mucosal hyperplasia and infiltration by leukocytes. Six MEs were evaluated for each strain (ECRG4^−/−^; WT) and time point.

### Gene array

Gene arrays were used to provide quantitative information on gene expression levels within the ME, since mRNA is extracted in a uniform manner from all cells in a tissue. Two experiments were performed first, and the MEs of WT mice were evaluated over the time course of acute OM. For this study, forty C57BL/6/WB F1 hybrid mice per time point were inoculated in the ME bilaterally with NTHi as mentioned above. Uninoculated animals served as pre-infection baseline control. Mucosal tissue and ME exudate were harvested from 20 mice at each of the following intervals: 0 h (0 h, uninfected), 3 h, 6 h, 1 day (1d), 2d, 3d, 5d, and 7d after inoculation, and pooled. The tissue was homogenized in TRIzol (Life Technologies, Carlsbad, CA), and total RNA was extracted, reverse transcribed, amplified, and transcribed *in vitro* to generate biotinylated cRNA probes that were hybridized to two Affymetrix (Agilent Technologies, Santa Clara, CA) MU430 2.0 microarrays, according to the manufacturer’s protocol. This procedure was duplicated for each time point to obtain a second, independent biological replicate. Thus, each data point represents two separate samples consisting of 20 mice each and four Affymetrix arrays. Specific genes were assessed at individual time points using Genespring GX 7.3 (Agilent Technologies, Santa Clara, CA). To identify changes in gene expression, the data were first analyzed using a variance modeling approach. The raw MAS5 expression values were imported into the VAMPIRE software without prior normalization. This program uses a Bayesian approach to identify significantly altered genes (Hsiao et al., 2005). Hybridization to probes for genes involved in AA metabolism and signaling were assessed at individual time points for difference from control (uninfected) samples, after Bonferonni correction for multiple tests and using ANOVA in Genespring GX 7.3 (Agilent Technologies, Santa Clara, CA). Additional details of methods are provided in our previous publication (Hernandez et al., 2015) in which report the genes evaluated here were not included. For the second experiment, groups of 20 mice each of C57 WT mice and *Ecrg4* KO mice on a C57 background were inoculated in the ME and identically treated as described above at 0 h, 6 h, and 2 days time points to identify genes differentially regulated by ECRG4. Mucosal tissue and ME exudate was then harvested from 20 mice at each of the time point intervals, RNA was extracted as mentioned before, and generated biotinylated cRNA were hybridized to two Affymetrix microarray chips. This procedure was duplicated for each time point to obtain a second, independent biological replicate for a total of four microarrays per condition and genotype. The raw MAS5 expression values were imported into the VAMPIRE software. The fold change (FC) was compared relative to genotype and time, and resulting *p* values were adjusted by Benjamini–Hochberg correction.

### Single-cell RNA-Seq

Gene arrays generated from bulk tissue cannot identify which cells are expressing the given gene. We used scRNA-Seq to provide precise, cell-level data. However, because the isolation of different cell types can vary in efficiency depending on the fragility and strength of bonding to other cells, this method is less able than gene arrays to determine overall ME levels of gene expression. Groups of six C57BL/6 mice each were untreated as controls. Additional groups made of six mice each were inoculated in the ME with NTHi and mucosal tissue and exudate harvested at 6 h, 1d, 2d, 3d, 5d, and 7d after inoculation. The pooled tissue for each sample was digested with thermolysin (0.5 mg/ml, Sigma-Aldrich, #T7902) followed by FACSMax cell dissociation solution (Genlantis, #T200100) and triturated into single cells. Dissociated cells were diluted to 700 cells/μL. Three replicates were performed to obtain independent biological samples of control MEs (Ryan et al., 2020), while four replicates were performed 24 h after NTHi inoculation. Other time points were made of three replicates each. Single-cell libraries were prepared using the Chromium Controller (10X Genomics, Pleasanton, CA) according to the manufacturers’ instructions. The libraries were sequenced on an Illumina HiSeq 2,500 (Illumina, San Diego), which yielded approximately 200 million reads per sample.

Bar-coded reads were demultiplexed using Cellranger 2.0.2 (10X Genomics) and mkfastq in conjunction with bcl2fastq 2.17.1.14 (Illumina) and aligned to a murine reference genome. Reads were filtered to remove short reads and reference genome mismatches to improve library quality, quantified, and subjected to principal component analysis (PCA) clustering. Identification of cells in each cluster was based on the following marker genes: epithelial cells, high expression of *Epcam and Krt18*; ciliated epithelial cells, *Epcam* and *Hydin*; basal epithelial cells, *Epcam and Krt5*; stromal cells, *Col1a2*; endothelial cells, *Egfl7*; pericytes, *Rgs5*; monocytes, *Csf1r*; lymphocytes, *Ptprcap*; and melanocytes, *Mlana*. After infection, PMNs were identified by the expression of *Stfa2l1* and red blood cells (RBCs) by *Hba-a1*. Graph-based and K-means analysis of gene expression was then performed for *Ecrg4* (150015O10Rik). The expression of the gene was visualized using 10X Genomics cLoupe. A PCA cluster plot for each gene was generated using log data. Violin plots of Ecrg4 expression levels for each cluster were log-normalized for optimal comparison across the cluster cell population. Additional details of methods are available in our previous publication on normal ME scRNA-Seq (Ryan et al., 2020).

### Western blotting and co-immunoprecipitation

Tissue lysates, from control or infected ME tissue, were run under reducing conditions on polyacrylamide gels and transferred to nitrocellulose membranes. Membranes were blocked with 5% BSA and then were probed with antibodies specific to ECRG4 [anti-Ecrg4 (1:2,500; HPA008546, Sigma), anti-Ecrg4 133–148 (1:1,000; G-012–24, Phoenix Pharmaceuticals)], and anti–β-actin (1:500; Cell Signaling Technologies). Four MEs (2 mice) were lysed with RIPA buffer supplemented with protease inhibitor cocktail (Pierce, Rockford, IL, United States of America) and dissociated through a 21 g needle. The insoluble fraction was pelleted at 10,000 × *g* and discarded. Soluble protein was pre-cleared with 2 μg of goat (for CD14) or rabbit (for TLR2, TLR4 and MD2) normal IgG and protein A/G agarose beads (Santa Cruz Biotech) for 1 h at 4°C with rotation. The IgG-bound proteins were centrifuged at 2,500 × *g* and discarded. Goat anti-CD14, rabbit anti-TLR2, rabbit anti-TLR4, and rabbit anti-MD2 (Santa Cruz Biotech) were each added at 2 μg and incubated overnight at 4°C with rotation. The following day, 20 μL of protein A/G beads were added and incubated for 1 h with rotation. Protein complexes were pelleted at 2,500 × *g* and washed three times with RIPA buffer. Protein was eluted by boiling in reducing 1X LDS sample buffer (Invitrogen) followed by centrifugation to pellet agarose beads. The immunoblotting protocol described above was then used to probe for ECRG4.

### Histology and morphometry

For histological analysis, mice were deeply anesthetized as mentioned above and sacrificed by decapitation at 1, 2, 3, 5, or 10 days after NTHi inoculation. One ME bullae was dissected, fixed overnight in 4% paraformaldehyde (PFA), decalcified in 8% EDTA in 4% PFA, embedded in paraffin, sectioned at 8 μm, and stained with hematoxylin and eosin. Control mice were not treated prior to sacrifice. Six mice were used for each time point. Mucosal thickness was measured at six standard locations in the ME and averaged to obtain a measure of mucosal hyperplasia.

The percent area of the ME lumen occupied by leukocytes was measured in six sections at the location of maximal leukocyte accumulation, and the numbers of PMNs and macrophages in high-magnification (400x) images of the cellular effusion were counted.

### Bacterial titers

The remaining bullae of the six mice used for histology were opened, and a 1 μL loop used to harvest ME fluid or to scrape the mucosal surface of dry ears. The samples were serially diluted and plated on chocolate agar plates, and bacterial titers were determined.

### Phagocytosis assay

Primary peritoneal macrophages were attained from six WT mice and six ECRG4^−/−^ mice by injection of 3 ml 4% thioglycolate media i.p., and 3 days later, we performed a peritoneal lavage with cold RPMI 1640 supplemented with 10% FBS, 50 U/ml penicillin, and 50 μg/ml streptomycin and β-mercaptoethanol. The isolated cells were then washed and counted before seeding into a 48-well plate at 5 × 10^5^ cells per well, in triplicate wells for each test condition. NTHi bacteria were then added at a count of 5 × 10^7^ cells per well. The plate was then centrifuged at 100 g for 5 min and allowed to incubate at 37°C. After 1 h, the extracellular bacteria were washed away with DMEM the contained gentamicin (50 mg/ml). The cells were then either lysed immediately to assess phagocytosis of NTHi or allowed to incubate for additional 3 h to evaluate intracellular killing. The macrophages were afterward rinsed with DMEM and lysed by adding 0.5 ml pyrogen-free distilled water followed by aspiration of the lysate five times through a 23-gauge syringe. Lysates were then diluted by 1:10 dilution up to 1:10^5^ and plated onto chocolate agar plates in serial dilution and incubated overnight at 37°C. The next day, colonies were counted to determine NTHi titers.

### Quantitative reverse transcription PCR

RNA was isolated from middle ear tissue using an RNeasy mini kit (Qiagen, United States) according to the manufacturer’s recommended procedures. Yields and purity were assessed using a NanoDrop2000 Spectrophotometer (Thermo Fisher Scientific). For gene expression analysis, the complementary DNA (cDNA) synthesis was accomplished using 1 μg of total RNA with an iScript (Bio-Rad) kit, according to the manufacturer’s protocol, and a final 200 ng of the cDNA was used for each real-time quantitative PCR determination. *Ecrg4* gene expression was probed with QuantiTect primer (no. QT00128051, Qiagen, Hilden, Germany), and data were normalized to glyceraldehyde-3-phosphate dehydrogenase (GAPDH) using the ΔΔ*C*_*T*_ method; primer was purchased from Qiagen (no. QT01658692). Melt curves were used to ensure a single amplicon amplification. Reactions were run on an iQ5 Real-Time PCR machine (Bio-Rad, Hercules, CA, United States) with the QuantiTect SYBR Green with the following parameters: 10 min at 95°C; 40 X (95°C for 30 s, 60°C for 30 s, and 72°C for 30 s); and finally, at 72°C for 2 min.

### Statistical analyses

Statistical analysis was conducted using StatView (JMP-SAS Institute, Cary, United States). Mucosal thickness, ME area occupied with leukocyte infiltrate, and numbers of neutrophil and macrophages were compared between *Ecrg4* KO and WT mice. Data are reported as mean ± SD. Differences were considered significant at *p* < 0.05. Two-tailed t-tests with Bonferroni correction for multiple comparisons were performed on measures of mucosal thickness and NTHi clearance. The Mann–Whitney *U*-test was used for data lacking a normal distribution, as in analyses of ME inflammatory cells. We evaluated the normality of the data by using the D’Agostino–Pearson omnibus test. Left and right ears in each mouse were considered to be independent of each other and, therefore, analyzed independently, as previously discussed in detail (Ebmeyer et al., 2005).

## Results

### ECRG4 down-regulation is inversely related to mucosal hyperplasia

**FIGURE 1:**
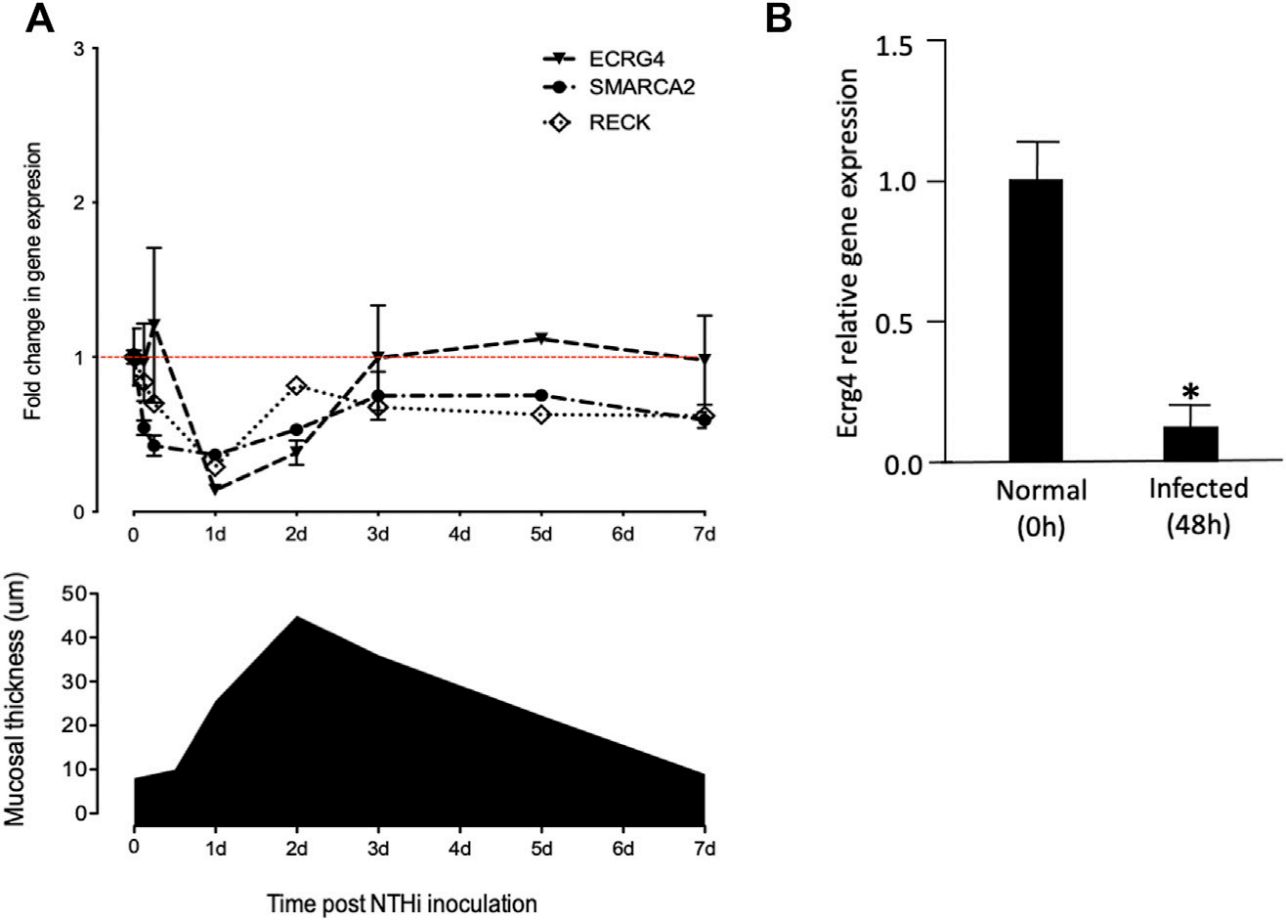
**(A)** Comparison of mucosal hyperplasia during the course of NTHi-induced OM in the mouse with gene array data on the expression of *Ecrg4*, *Reck*, and *Smarca2*. Thickness data represent 6 MEs at each time point. Array data for each time represent two samples of 20 mice, each hybridized to two arrays. Detailed array data are presented in Table 1. Red line features the baseline gene expression at day 0. **(B)** qPCR of middle ear tissue confirming the down-regulation of *Ecrg4* expression 48 h after infection.

To evaluate the potential role of growth suppressor genes in OM, in our gene microarray database of a complete episode of murine OM, we assessed the expression of 79 genes in the Gene Ontology (GO) categories “negative regulation of cell proliferation,” “negative regulation of the cell cycle,” and “negative regulation of cell growth” and 637 tumor suppressor genes in the TSGene database (Zhao et al., 2016). We used Genespring for array data (Hernandez et al., 2015) and 10X Cellranger for scRNA-Seq data visualization. In particular, we searched for growth suppression genes that were down-regulated with a temporal relationship to mucosal hyperplasia during an episode of acute OM. Three genes were identified as exhibiting strong (50% or greater) down-regulation restricted to 6 h, 1d, and 2d (Figure 1A and Table 1): *Ecrg4* (86% down-regulation), *Reck* (74% down-regulation), and *Smarca2* (50% down-regulation). *Ecrg4* is a tumor suppressor potentially involved in esophageal cancer (Yue et al., 2003). *Reck* is a tumor suppressor involved in tumors at many sites, including middle ear squamous cell carcinoma (Liu et al., 2012). *Smarca2* is a regulator of chromatin structure (Guerro-Martinez and Reyes, 2018).

**TABLE 1:** Genes down-regulated in the temporal relationship to mucosal hyperplasia in WT mouse ME gene arrays.

Time after ME infection	Fold change	Fold range	*p* value
*Ecrg4* 1440780_x_at			
Time 0 h	1.000	(0.97–1.03)	0.991
Time 3 h	0.950	(0.789–1.143)	0.827
Time 6 h	1.155	(0.853–1.564)	0.718
Time 1 d	0.140	(0.135–0.145)	0.012
Time 2 d	0.379	(0.326–0.439)	0.097
Time 3 d	0.966	(0.755–1.235)	0.910
Time 5 d	1.117	(1.089–1.145)	0.144
Time 7 d	0.958	(0.776–1.183)	0.873
*Smarca2* 1430526_a_at			
Time 0 h	0.983	(0.814–1.186)	0.941
Time 3 h	0.542	(0.497–0.591)	0.089
Time 6 h	0.422	(0.361–0.493)	0.114
Time 1 d	0.368	(0.345–0.392)	0.041
Time 2 d	0.531	(0.507–0.556)	0.047
Time 3 d	0.735	(0.596–0.906)	0.380
Time 5 d	0.752	(0.738–0.766)	0.042
Time 7 d	0.591	(0.542–0.644)	0.103
*Reck* 450784_at			
Time 0 h	0.982	(0.813–1.187)	0.940
Time 3 h	0.836	(0.772–0.906)	0.269
Time 6 h	0.651	(0.441–0.962)	0.470
Time 1 d	0.289	(0.256–0.325)	0.060
Time 2 d	0.816	(0.811–0.822)	0.023
Time 3 d	0.658	(0.519–0.834)	0.328
Time 5 d	0.628	(0.608–0.648)	0.043
Time 7 d	0.527	(0.292–0.95)	0.473

RECK, given its role in ME neoplasm, was of interest. However, squamous cell carcinoma at this site appears to be epidermal in origin, reflecting invasion of the ME by skin cells (Liu et al., 2012). Moreover, RECK regulates MMP9 and exerts its effects primarily through suppression of WNT7A/WNT7B (Cho et al., 2017), but regulation of neither *Wnt7a* nor *Wnt7b* regulation was observed in our gene microarray dataset, and scRNA-Seq did not detect *Wnt7a* nor *Wnt7b* expression, before or after infection, in any ME cells. As a chromatin modifier, SMARCA2 seems most likely to be downstream from cell signaling events controlling mucosal hyperplasia. We, therefore, concentrated upon ECRG4.

ECRG4’s growth suppression potential was discovered due to down-regulation in 15 out of 20 human esophageal epithelial cell cancers (Yue et al., 2003). As noted above, and in earlier study (Kurabi et al., 2013), *Ecrg4* expression exhibited a strong decrease beginning 6 h after NTHi inoculation of the ME, which recovered to pre-infection levels by 3d. Increase in ME mucosal thickness showed a similar time course. This early decrease in *Ecrg4* gene expression, being lowest at 1 day, preceded the maximal increase in epithelial mucosal thickness observed, which peaked at 2 days after NTHi infection. The decrease in ECRG4 mRNA expression was confirmed by a comparison of qPCR data from control versus 48 h after ME NTHi infection (Figure 1B).

### ECRG4 is primarily expressed by stromal cells in the ME

**FIGURE 2:**
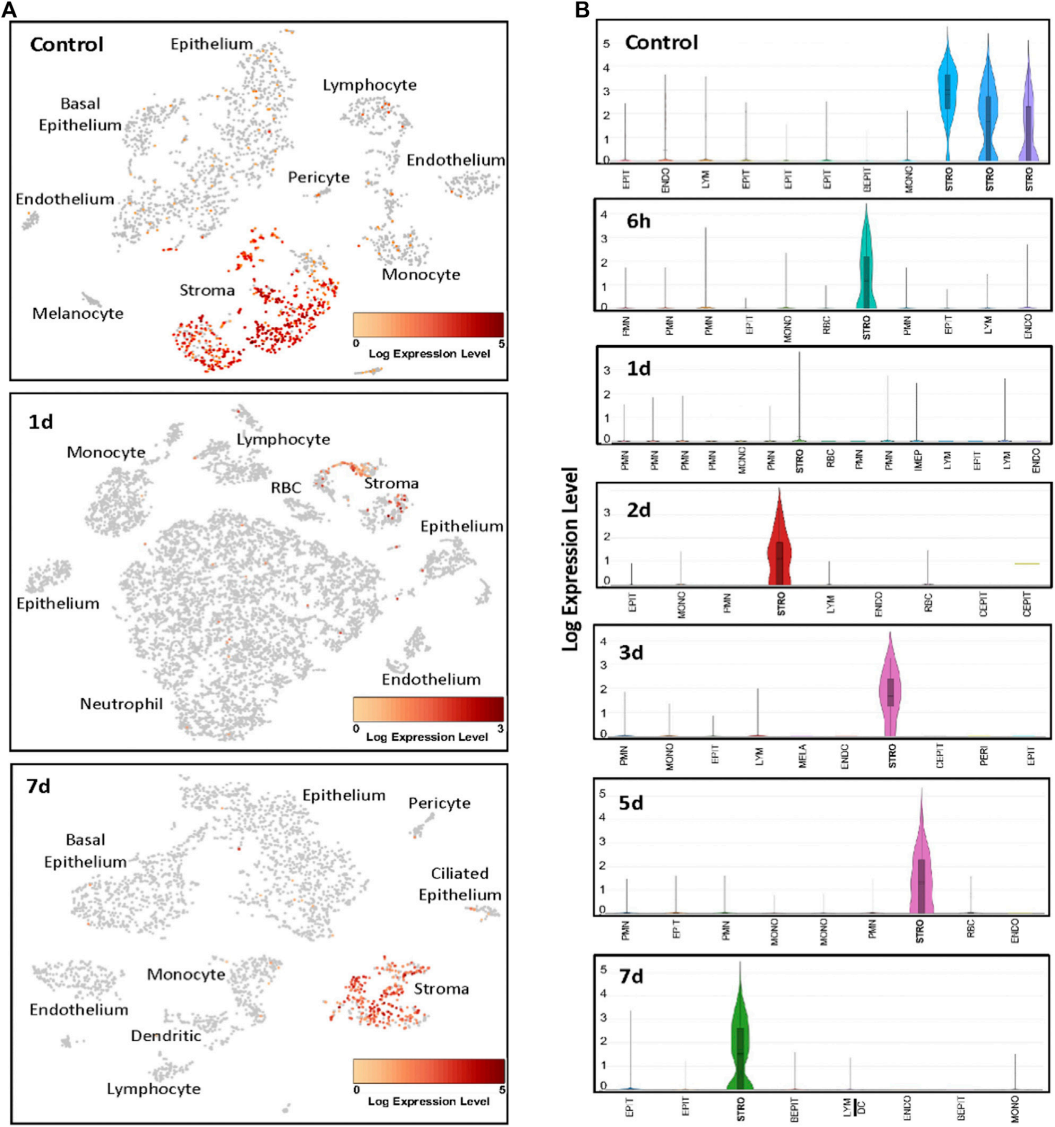
Examples of scRNA-Seq *Ecrg4* expression during the course of NTHi-induced murine OM. **(A)** Cells were clustered by PCA, and cell types of each cluster were determined by known marker gene expression (Ryan et al., 2020). Cells expressing *Ecrg4* are indicted in red. **(B)** Violin plots of the individual PCA clusters were identified by cell type, with some cell types being represented by multiple clusters. The width of every plot represents the frequency of cells expressing the gene at that log-normalized level. The upper horizontal line on each plot represents the cluster mean, and the lower line the median. The height of the vertical line represents the highest expressing cell. Each sample presented represents MEs from six mice. EPIT, epithelium; BEPIT, basal epithelium; IEPIT, intermediate epithelium; CEPIT, ciliated epithelium; STRO, stroma; ENDO, endothelium; PERI, pericyte; MONO, monocyte; DC, dendritic cell; LYM, lymphocyte; PMN, polymorphonuclear leukocyte/neutrophil; and RBC, erythrocyte.

ScRNA-Seq was used to identify the cells expressing *Ecrg4* during acute murine OM. The results are illustrated with examples of individual ME samples analyzed by 10X Genomics cLoupe, in Figure 2 and Table 2. Marker genes employed for normal ME and other additional details of methods can be found in the work of Ryan et al. (2020). In the NTHi-infected ME, PMNs were identified by the expression of *Il1f9* and *Stfa2l1,* while red blood cells were identified by hemoglobin gene expression (*Hba-a1*). The expression of genes in the various cell type clusters was visualized using cLoupe (10X Genomics) violin plot displays.

**Table 2:** Stromal cell scRNA-seq *Ecrg4* normalized expression level after ME infection.

Time	Mean	Median	Minimum–maximum range
Time 0 h	1.79	1.58	0–5.43
Time 6 h	1.52	1.00	0–5.09
Time 1 day	0.14	0.00	0–3.91
Time 2 days	1.61	1.58	0–4.52
Time 3 days	2.59	2.70	0–5.46
Time 5 days	2.56	2.00	0–6.61
Time 7 days	1.52	1.00	0–5.13

In the healthy normal ME sample, PCA analysis identified three classes of stromal cells using the marker *Col1a2* (Ryan et al., 2020). We reanalyzed the normal ME dataset from a previous study (Ryan et al., 2020) for gene/feature expression by graph-based and K-means using cLoupe and found that *Ecrg4* was expressed by all three. All other cell types showed weak expression by only a small minority of cells. After NTHi inoculation, PCA grouped all stromal cells into a single cluster. At 6 h after infection, the normalized *Ecrg4* expression level by stromal cells had decreased slightly, but by day 1, it was down to 86% (Figure 2) matching the gene microarray data which indicated maximum regulation of *Ecrg4* expression. On day 2, stromal expression level had partially recovered, and by day 3, it was fully restored. Figure 1A shows that on day 7, *Ecrg4* expression level is recovered to basal level. Thus, the scRNA-Seq confirms and validates the microarray expression profile of *Ecrg4* and shows that it is localized mainly to stromal cells since *Ecrg4* expression by all other cell types was limited to none or a small fraction of cells.

### ECRG4 is enzymatically processed during OM

**FIGURE 3:**
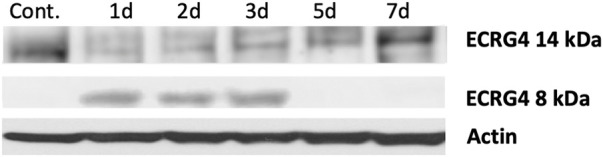
Western blot of mouse middle ear mucosa probed for ECRG4, before and after NTHi infection, using an antibody to the extracellular portion. Prior to infection (control, 0 h), only the full-length form is detected. After infection, the 8 kDa form augurin is present. Each blot represents the MEs of six mice.

ECRG4 is a transmembrane protein that can be enzymatically cleaved by thrombin to release its 8 kDa extracellular domain as a soluble molecule known as augurin, a mediator with diverse functions including regulation of tissue growth and macrophage activation (Gonzalez et al., 2011; Baird et al., 2012). Western blot analysis of middle ear tissue for ECRG4 protein (Figure 3) showed expression only of the full-length, 14 kDa form prior to middle ear infection. After infection, the full-length form was no longer present, and the 8 kDa form was predominant.

### Lack of ECRG4 alters mucosal hyperplasia during OM

**FIGURE 4:**
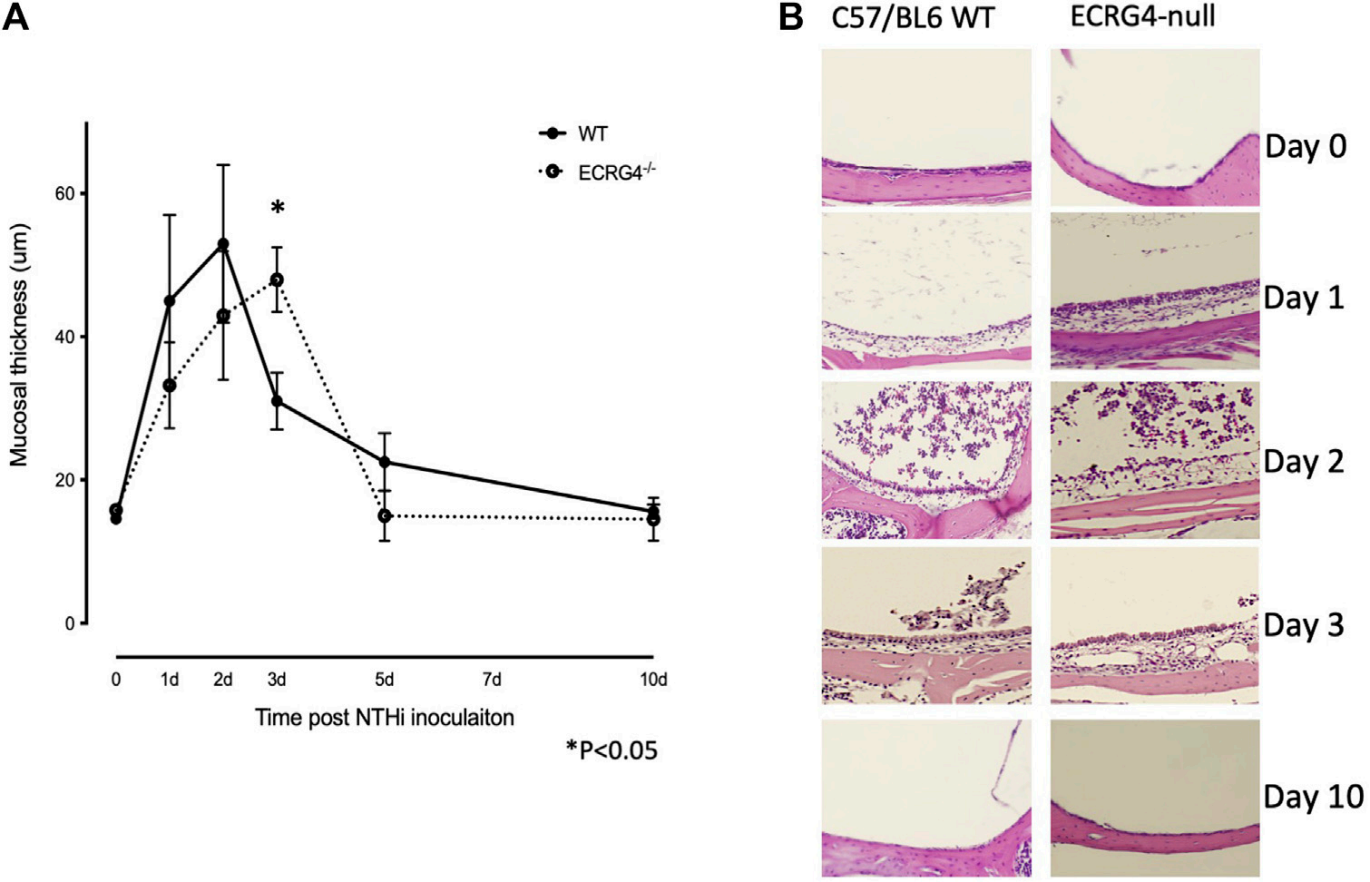
**(A)** Quantitative measurements of the middle ear mucosal thickness over the time course of experimental OM in WT and *Ecrg4* ^−/−^ mice. Quantitative data representing 6 MEs for each data point. **(B)** Representative histological examples of the ME mucosa, before and during NTHi-induced OM, in WT and *Ecrg4* ^−/−^ mice at identified times.

We compared the histology of WT and ECRG4-deficient mice during the course of NTHi-induced acute OM. As shown in the examples presented in Figure 4A, mucosal thickness was similar in the MEs of *Ecrg4*−/− and WT mice, with the exception of day 3, when thickness was significantly greater in the KO (Figure 4B).

### Lack of ECRG4 increases the number of macrophages in the middle ear during OM

**FIGURE 5:**
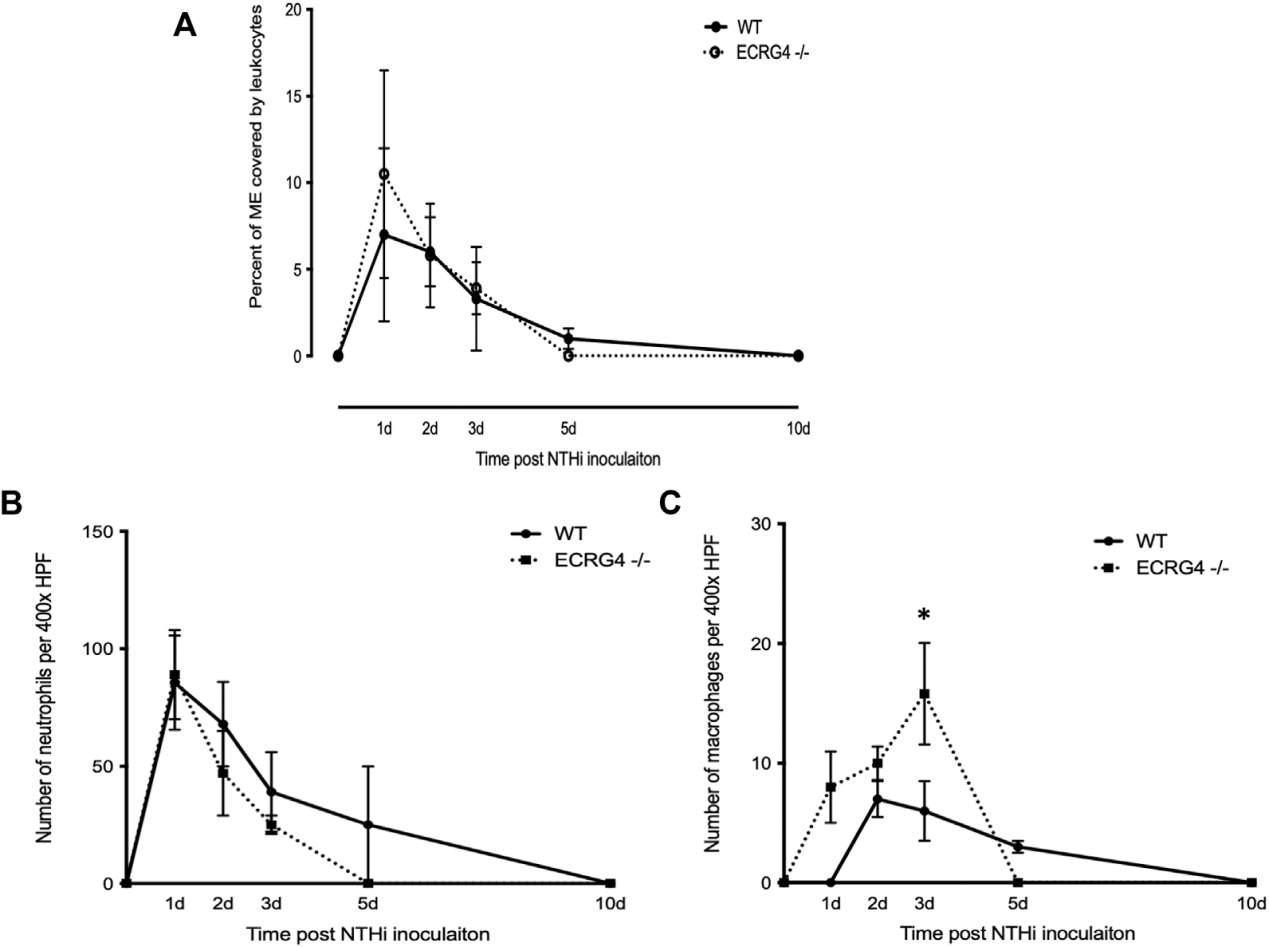
**(A)** Percent area of the middle ear occupied by leukocytes during the course of NTHi-induced OM in WT versus *Ecrg4* ^−/−^ mice. **(B)** Number of PMNs counted in a 400x field of the ME cellular effusion. **(C)** Number of macrophages counted in a 400x field of the ME cellular effusion.

Mice deficient in ECRG4 showed recruitment of leukocytes into the ME lumen, assessed as the percent of the middle ear lumen occupied by leukocytes, which was comparable to that in WT mice (Figure 5A). The numbers of PMNs counted within the cellular effusion were also similar, although there was a non-significant trend for earlier clearance (Figure 5B). In contrast, macrophages not only entered the middle ear earlier in ECRG4-null mice and were greater in number but also cleared earlier than in WTs (Figure 5C).

### Middle ear bacterial clearance and macrophage phagocytosis are enhanced in *ECRG4* ^−/−^ mice

**FIGURE 6:**
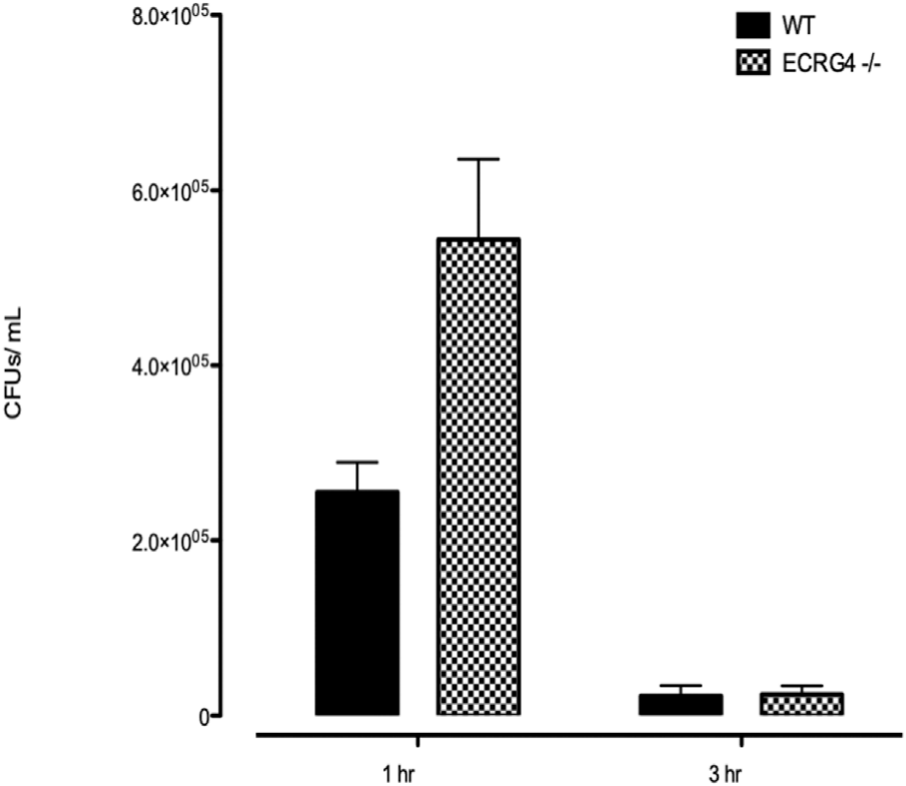
*In vitro* phagocytosis and intracellular killing of NTHi in peritoneal macrophages harvested from WT versus *Ecrg4* ^−/−^ mice.

ME NTHi culture positivity was compared between WT versus *Ecrg4* KO mice during OM (Table 3). *Ecrg4* ^−/−^ mice cleared bacteria earlier than WT mice. Peritoneal macrophages from ECRG-null mice exhibited enhanced NTHi phagocytosis *in vitro*. However, the KO mice were able to eliminate the higher number of intracellular NTHi, perhaps indicating enhanced intracellular killing (Figure 6).

**TABLE 3:** Middle ear NTHi culture positivity for WT versus *Ecrg4* knockout mice represented by colony forming units (CFUs). *Ecrg4* KO mice cleared more rapidly than WT mice. Data represent culture positive plates out of six.

Time after NTHi inoculation	C57 WT number of culture positive ears from total ears	C57 WT mean CFUs of culture-positive plates	ECRG4^−/−^number of culture-positive ears from total ears	ECRG4^−/−^ mean CFUs of culture-positive plates
Day 0	0 : 6	0.00	0 : 6	0.00
Day 1	4 : 6	>10^4^	6 : 6	>10^4^
Day 2	6 : 6	>10^4^	4 : 6	>10^4^
Day 3	3 : 6	∼600	0 : 6	0.00
Day 10	0 : 6	0.00	0 : 6	0.00

### Lack of ECRG4 alters middle ear gene expression

**FIGURE 7:**
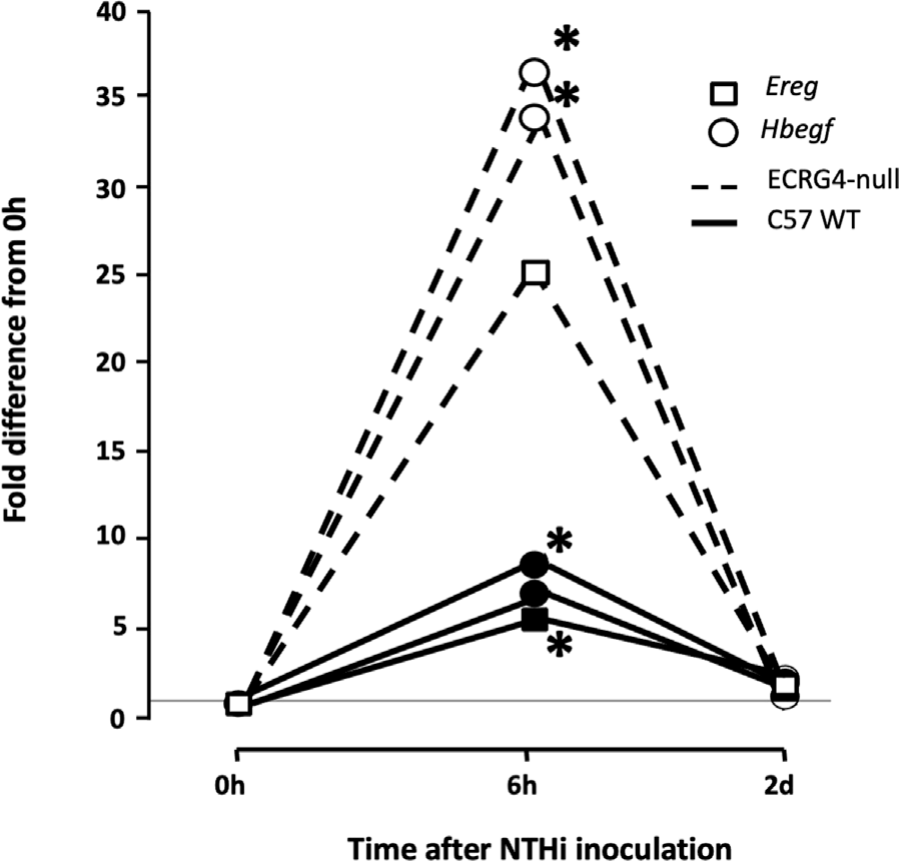
Gene array data comparing fold change in expression from 0 h, at 6 h, and 2d after NTHi inoculation of *Hbegf* and *Ereg* in WT mice versus mice deficient of the *Ecrg4* gene. At 6 h, the expression of two different probes for *Hbegf,* compared to that at 0h, is up-regulated more than 30-fold. In comparison, expression in WT mice at 6 h is up-regulated only 6–8-fold. Similarly, an *Ereg* probe is up-regulated by 25-fold at 6 h, compared to 5-fold in the WT. By 2 days after ME infection, expression in both genotypes has recovered to near pre-OM levels. The horizontal line across indicates baseline 0 h fold difference.

Gene array analysis of OM between WT and ECRG4-null mice for genes with a significant interaction across genotype and infection identified 1,277 differentially expressed genes. Among the 50 most significantly different genes (Table 4), 21 were related to tissue growth, 12 were related to protein processing, and 5 to mitochondrial function, accounting for three-quarters of the total. To identify the genes most strongly regulated in the full dataset, we evaluated all genes that exhibited a 5-fold or greater difference in expression at each time, which totaled 84 (Table 5). Of the eleven genes that exhibited a 5-fold or greater difference in expression between the genotypes at 0 h, six were related to tissue growth and two to immune response. The majority were expressed at a lower level in the KO. At 6 h, of the 56 genes regulated more than 5-fold, 16 were related to tissue growth and 31 to immune response. Most of these genes were up-regulated in the KO. At 2 days, of the seventeen genes regulated >5-fold, six genes were related to tissue growth and five to immunity. Most of the 2-day >5-fold genes were again down-regulated. Of the total 84 genes, nearly half (38) were related to immune response and 28 to tissue growth. Interestingly, both the *Hbegf* gene that we have strongly linked to mucosal growth (Suzukawa et al., 2014; Sakamoto et al., 2022) and the gene for epiregulin (*Ereg*), which also activates the same EGF receptors, were far more highly up-regulated at 6 h after NTHi infection in the ECRG4-null middle ear than in WTs (Figure 7).

**TABLE 4:** Top 50 regulated genes arranged by *p* value between WT vs. *Ecrg4* ^*−/−*^ middle ear gene arrays for genotype and time. FC, fold change [bold, tissue growth related (20 genes); underline, immune response related (13 genes); and * = protein synthesis/degradation related (7 genes)].

Gene	FC	*p* value	Gene	FC	*p* value
***Pitx2***	2.53	2.4E-06	*Ap2m1*	2.32	7.1E-06
Sae1*	1.63	3.8E-06	*Myoz1*	2.46	7.1E-06
*Mknk1*	3.60	3.8E-06	*Pkm*	2.71	7.1E-06
Tcean2*	2.35	3.8E-06	***Hspb8***	2.35	8.0E-06
***Mob1a***	2.08	3.8E-06	*Tspan17*	1.24	8.2E-06
*Dpp8*	2.37	3.8E-06	*Nsun5*	2.77	8.2E-06
Asb6*	1.77	3.8E-06	***Lin37***	2.62	9.9E-06
***Enah***	−5.03	3.8E-06	*Fzd6*	1.86	1.1E-05
Copa*	1.61	4.5E-06	***Mapre1***	1.39	1.1E-05
***Sdc4***	−62.12	4.5E-06	*Ppp2r5c*	2.95	1.1E-05
*Rad51l*	1.70	4.5E-06	*Rexo4*	2.64	1.1E-05
***Angel2***	2.93	4.5E-06	*Atp5gr*	5.06	1.1E-05
Arf4*	2.47	4.6E-06	***Kalrn***	4.05	1.1E-05
*Atrn*	1.53	4.8E-06	*Rnf19a*	1.55	1.1E-05
***Mad1l1***	2.69	5.6E-06	***Arf1***	1.74	1.1E-05
***Wdr55***	3.22	5.6E-06	Eif4b*	1.46	1.1E-05
***Dusp10***	3.01	6.2E-06	Rad23a*	1.82	1.1E-05
*Sephs2*	5.93	6.8E-06	***Cxcl16***	1.56	1.2E-05
*Mtg2*	2.16	7.0E-06	*Abcc1*	1.58	1.2E-05
***Prkd3***	3.25	7.1E-06	*Vamp3*	1.65	1.2E-05
*Kcnma1*	−5.08	7.1E-06	*Esyt3*	−2.02	1.2E-05
***Os9B***	3.45	7.1E-06	*Kif1*	1.40	1.3E-05
***Cilp2***	2.42	7.1E-06	*Tial1*	2.75	1.3E-05
***Mxd4***	1.46	7.1E-06	***Cxx1a***	1.86	1.3E-05
*Mid2*	1.87	7.1E-06	***Ikbkb***	2.86	1.3E-05

**TABLE 5:** Genes with a >5-fold difference between WT and *Ecrg4* ^*−/−*^ mice middle ear in gene arrays. (**bold**, cell growth; underline, immune related). Positive values indicate higher levels in WT ME.

0h			6h			48h		
Gene	FC	*p* value	Gene	FC	*p* value	Gene	FC	*p* value
***Csnk1d***	5.39	1.9E-05	*Akr1b8*	6.80	6.7E-04	*Abca1*	5.80	3.1E-05
*Ddx3y*	−32.57	2.9E-04	*Ankrd1*	5.43.	1.3E-04	*Col1a1*	6.70	5.2E-05
***Malat1***	7.63	0.0036	***Apol9b***	11.75	5.8E-04	*Ddx3y*	-30.79	2.8E-04
*Morn3*	6.09	1.8E-05	*Atp5g2*	5.06	1.1E-05	*Dhx58*	7.20	2.7E-05
*Os9*	5.26	7.1E-06	*BC023105*	5.79	8.4E-04	***Epb4.1l***	6.07	3.3E-05
***Ppp2r5c***	6.39	1.1E-05	*Brix1*	5.26	6.4E-04	***Grb10***	6.00	1.7E-05
**Prkd3**	6.28	7.1E-06	*Ccl11*	6.21	0.00130	*Gusb*	5.00	6.0E-05
**Prr15**	−8.00	2.1E-05	*Cfb*	6.91	0.00133	*Igha*	-11.02	0.0167
**Ptpn13**	5.40	1.1E-04	*Cxcr6*	5.77	0.00129	*Il4ra*	6.27	2.1E-05
*Sdc4*	−13.12	4.5E-06	*Cyb7p1*	5.23	0.00497	***Mknk1***	7.10	0.0146
*Slc32a2*	6.30	2.2E-05	***Dhx58***	14.24	2.7E-05	*Myh*	-6.93	4.2E-04
***Ywhaz***	5.81	9.2E-05	*Eif2ak2*	9.71	2.4E-05	Pla2g15	5.85	1.8E-04
			***Ereg***	-7.07	4.0E-04	***Prkd3***	7.58	7.0E-06
			***Fosb***	-16.41	4.2E-04	*Rab31*	5.78	1.3E-05
			**Ggct**	5.80	4.4E-04	*Scpep1*	5.53	2.0E-05
			GM14085	7.09	1.1E-04	***Sdc1***	5.02	2.1E-05
			GM16340	10.56	3.1E-04	*Sdc4*	-31.60	4.47E-6
			GM20559	8.11	5.9E-04	*Tnfsf14*	5.04	3.9E-05
			Gzma	5.96	3.3E-04			
			Ido1	7.00	0.00139			
			Ifi44	7.20	3.8E-04			
			Ighg2c	15.56	0.00435			
			Iglv1	11.78	0.03216			
			Kif1b	5.45	1.3E-05			
			Lgals9	5.24	2.2E-05			
			Lipg	8.48	8.3E-04			
			**Lipt1**	5.41	9.5E-05			
			**LOC1005039**	7.10	2.7E-04			
			**Malat1**	-6.25	2.9E-05			
			Ms4a4b	8.19	0.00137			
			**Myl1**	7.28	1.1E-04			
			Nmi	5.68	2.6E-04			
			Noxo1	5.39	2.2E-04			
			Oas1a	6.72	0.00109			
			Oas3	7.14	0.00131			
			Parp9	5.54	5.88E-4			
			*Pdcd1*	5.03	5.9E-04			
			**Plac8**	5.00	0.00190			
			Prg4	5.50	0.00108			
			**Psat1**	5.08	7.40E-4			
			Pus3	5.15	1.8E-04			
			*Sdc4*	-62.12	4.5E-06			
			Sectm1b	6.99	0.00124			
			Sephs2	5.93	6.8E-06			
			Serpina3n	8.78	0.00102			
			Slc28a3	8.23	2.1E-04			
			**Sp100**	5.10	3.1E-05			
			Tlr3	6.31	5.2E-05			
			*Tnfsf14*	6.40	1.7E-04			
			Tor3a	7.02	1.5E-04			
			**Tpx2**	9.20	5.2E-05			
			Trim21	5.30	5.7E-05			
			**Trim12a**	7.10	9.9E-05			
			Trim30d	18.90	1.3E-05			
			**Trpm7**	-8.49	4.7E-05			
			**Zfp322a**	5.70	7.4E-04			

### Augurin binds the LPS receptor in the middle ear

**FIGURE 8:**
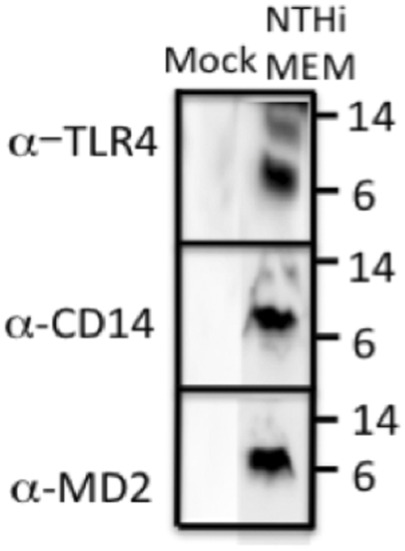
Western blot of ECRG4 immunoprecipitation in ME protein, probed with antibodies against TLR4, CD14, or MD2, showing binding of augurin to the elements of the LPS receptor complex.

The fact that reduced ECRG4 altered macrophage infiltration and phagocytosis in the middle ear suggested a relationship between ECRG4 and inflammation/innate immunity. Podvin et al. (2015) found that augurin binds to the endotoxin receptor, after which it is internalized. To probe this possibility in the ME, we co-immunoprecipitated ECRG4 with molecules involved in innate immunity in the middle ear mucosa during OM (TLR2, TLR4, CD14, and MD2). We confirmed that the ∼8 kDa fragment of ECRG4, the only form present in the middle ear during OM, immunoprecipitated with antibodies against TLR4, its co-receptor CD14, and MD2, which together form the lipopolysaccharide (LPS) receptor (Figure 8). TLR2 co-immunoprecipitation was negative (not shown).

Bacterial LPS receptor signals *via* TLR4 and the adaptor MYD88 to regulate, among others, interleukin gene expression and *via* TLR3 and the adaptor TRIF to regulate interferon genes. To determine whether lack of ECRG4 influenced genes related to these LPS targets, we assessed the gene array data for differential expression of interleukin- and interferon-related genes between *Ecrg4* ^−/−^ and WT mice during OM. We found that 31 interleukin-related genes (7 more than 2-fold) and 13 interferon-related genes (7 more than 2-fold) were significantly regulated, including both ligand and receptor genes (Table 6). The great majority were up-regulated in the absence of ECRG4, suggesting release from inhibition.

**TABLE 6:** Interleukin- and interferon-related genes differentially regulated (>2-fold) in WT versus the *Ecrg4* ^*−/−*^ mice from ME gene arrays. Positive values indicate higher expression in WT ME.

Interleukin-related genes			Interferon-related genes		
Gene	FC (6 h)	*p* value	Gene	FC (6 h)	*p* value
*IL11*	3.03	9.9E-05	*Ifnb1*	−2.04	0.0410
*Il12b*	−2.19	0.0011	*Ifnar2*	2.33	5.8E-04
*Il15*	4.97	7.4E-04	*Ifngr2*	2.03	1.6E-04
*Il22*	2.56	0.0369	*Irf3*	2.28	1.3E-04
*Il1r2*	2.74	0.0202	*Irf5*	2.09	0.0039
*Il1rl1*	2.62	0.0197	*Irf7*	8.23	0.0022
*Il1rn*	5.06	0.0040	*Irf9*	2.52	3.8E-04
*Il1f9*	5.91	0.0163			
*Il1rap*	4.06	7.8E-04			
*Il2ra*	2.51	2.8E-04			
*Il2rg*	2.25	2.3E-04			
*Il4ra*	2.13	1.7E-04			
*Il4i1*	4.31	5.2E-04			
*Il15ra*	2.04	5.4E-05			
*Il17ra*	2.55	0.0033			
*Il13ra1*	3.09	8.0E-05			

To identify potential LPS target cells in the ME, we assessed scRNA-Seq data (Supplementary Table S1). This analysis showed that the expression of *Tlr4*, minimal prior to middle ear infection, was seen primarily in the epithelium, endothelium, and PMNs at 6 h, epithelium, monocytes/macrophages, and PMNs at 1d, and restricted to monocytes by 2d. *Cd14* was expressed by the epithelium, endothelium, and monocytes/macrophages before OM, with the endothelium and PMNs added after infection. The MD2 gene *Ly96* was expressed prior to infection in the endothelium, stroma, and monocytes/macrophages. At 6 h after infection, it was present primarily in the epithelium, endothelium stroma, and PMNs; at 1d, in the epithelium, endothelium, monocytes/macrophages, and PMNs; and at 2d, in the epithelium, monocytes/macrophages, and PMNs.

It has also been shown that augurin binds to the LDL receptor ORL1 (LOX-1) and the scavenger receptors SCARF1, CD36, and STAB1 (STABILIN-1), which also serve as pattern recognition receptors in innate immunity, activating NfκB in a MyD88-dependent fashion (Moriguchi et al., 2018). To identify potential augurin targets, we also assessed scRNA-Seq data (Supplementary Table S1). We found that *Orl1* mRNA, not expressed prior to middle ear infection, was observed in monocytes/macrophages and PMNs at 1d after infection and only in monocytes/macrophages at 2d. *Scarf1* mRNA was present only in the endothelium both before and 6h to 2d after infection. *Cd36* was expressed in the epithelium and monocytes/macrophages prior to and 6 h after infection, but only monocytes/macrophages at 1d and 2d. *Stab1* was present in the endothelium and monocytes/macrophages before infection and 1d and 2d OM, but only in the endothelium at 6 h. Taken together, these observations identify many middle ear cells that could be influenced by ECRG4 during OM.

## Discussion

We found that the expression of ECRG4, a 14 kDa transmembrane protein with a large extracellular domain, is localized to stromal cells in the ME. After infection, the *Ecrg4* gene expression is suppressed (Figures 1, 2; Table 1 and 2) and the existing protein is almost completely cleaved to release the extracellular domain as the soluble molecule augurin (Figure 3). Lack of full-length ECRG4 and the presence of augurin are correlated with prolonged middle ear mucosal hyperplasia (Figure 4). OM in ECRG4-null mice is also characterized by enhanced macrophage entry into the middle ear (Figure 5) phagocytosis (Figure 6) and increased expression of the growth factor genes *Hbegf* and *Ereg* (Figure 7), as well as more rapid clearance of bacteria (Table 3).

As a tumor suppressor gene, full-length ECRG4 may act to block the growth of stromal cells in the normal ME, maintaining homeostasis of the mucosa. Inflammation-induced cleavage of ECRG4, coupled with repression of gene expression to prevent replacement of the full-length form, would be expected to release this inhibition, allowing stromal expansion. Moreover, in other tissues, augurin has been shown to mediate cellular proliferative responses (Gonzalez et al., 2011). This raises the possibility that augurin may also stimulate growth in the mucosal stroma, epithelium, and/or endothelium and prolong hyperplasia. This possibility is supported by the presence of augurin receptors on these cell types (Supplementary Table S1).

Lack of ECRG4 had profound effects on innate immune function, in particular enhancing the entry and activity of macrophages. Since we did not observe pronounced expression of the *Ecrg4* gene in monocytes/macrophages (Figure 2), this must reflect an indirect effect. It is possible that lack of ECRG4 or the release of augurin alters the production of macrophage chemokines or activating factors by stromal and other middle ear cells. Since augurin binds to the LPS receptor complex in the infected middle ear and mice lacking ECRG4 express higher levels of interleukins, interferons, and their receptors, it seems most likely that augurin acts to inhibit responses to LPS, reducing innate immune stimulation of cells bearing this complex. Since augurin induces internalization of the receptor (Gonzalez et al., 2011), it may reduce the amount available for LPS stimulation. During OM, the LPS receptor elements are observed in many middle ear cell types. Released inhibition of LPS activation would be consistent with enhanced expression of inflammatory mediators and more rapid clearance of bacteria in ECRG4^−/−^ middle ears as well as enhanced activation of ECRG4-null macrophages. Paracrine effects of augurin during OM may also be mediated by interaction with ORL1, SCARF1, CD36, and/or STAB1 to influence various middle ear cell types. The many immune-related genes down-regulated by lack of ECRG4, especially at 6 h after infection (Tables 5, 6), also indicate a major role in regulating immunity.

Changes in growth factor and other growth-related gene expression before and during OM in ECRG4 KO mice suggest that ECRG4 is a major negative regulator of middle ear tissue growth in OM. Many of the genes differentially regulated in WT versus ECRG4^−/−^ mice are involved in cell replication and/or differentiation, and by 6 h after infection, the expression of the majority of growth regulators substantially increased in the *Ecrg4* ^−/−^ middle ear (Tables 4, 5). In particular, negative regulation of *Hbegf* and *Ereg*, both of which are strongly up-regulated by NTHi in the middle ear, implicates ECRG4 and augurin in the growth of the mucosa because the EGFR receptor for both growth factors is expressed in the ME mucosal epithelium and stroma. ECRG4 may also regulate its own expression. Dang et al. (2017) found that Sp1 is a positive regulator of the *Ecrg4* gene. We found that the Sp1 gene is expressed at a significantly lower level (FC = -3.59) in the uninfected Ecrg4^−/−^ ME, when compared to WT. After infection, the expression difference was lower at 6 h (FC = -2.87) and approached the WT value (FC = -1.51) at 2d. Since ECRG4 expression declines in the WT over this same period, this suggests a potential positive feedback loop for ECRG4.

The behavior of ECRG4, therefore, appears to be that of a sentinel protein that primarily inhibits proliferative and inflammatory reactions in the ME. Release from this inhibition by cleavage of full-length ECRG4 stimulates stromal hyperplasia and releases augurin, which can potentially affect many middle ear cell types with augurin receptors. The results of this study suggest that ECRG4 could be an attractive therapeutic target which can be used to reduce both inflammation and prolonged mucosal hyperplasia during OM.

Since our study utilized gene arrays, it is important to acknowledge some of the methodological limitations. As noted in methods, we had small number of biological replicates for each of the analyses of WT and *Ecrg4* ^−/−^ mice middle ear tissue. However, in the analysis to identify differentially expressed genes, we utilized both Bonferonni and Benjamini–Hochberg corrections for controlling the false discovery rate (Jørstad et al., 2007; Jafari and Ansari-Pour, 2019).

## Data Availability

The data presented in the study are deposited in the Science Data Bank repository, accession number 10.57760/sciencedb.01953. The link http://cstr.cn/31253.11.sciencedb.01953.
