# Supplementary material for: The ECRG4 cleavage product augurin binds the endotoxin receptor and influences the innate immune response during otitis media
Source: Front Genet. 2022 Aug 26;13:932555. doi: 10.3389/fgene.2022.932555 (PMC9461705; doi:10.3389/fgene.2022.932555)
Supplement: Supplementary file 1 [file DataSheet2.PDF]

Raw gene array values for genes regulated more than 5-fold in the ECRG-null middle ear (Table 5)

**0h**	**0h C57 raw**	**0h ECRG4 KO raw**
1. Csnk1d	1107.9287	146.11678
2. Ddx3y	9.431005	247.95978
3. Malat1	1368.0885	127.079605
4. Morn3	591.27893	67.030655
5. Os9	683.43365	91.88911
6. Ppp2r5c	418.39923	43.831093
7. Prkd3	198.39629	20.425827
8. Prr15	15.549713	92.76644
9. Ptpn13	137.60255	17.02956
10. Sdc4	367.1247	3364.8684
11. Slc23a2	252.1295	25.914837
12. Ywhaz	651.6234	78.54468

**6h**	**6h C57 raw**	**6h ECRG4 KO raw**
1. Akr1b8	3932.4446	402.33517
2. Ankrd1	127.288826	16.915295
3. Apol9b	563.7733	32.14441
4. Atp5g2	94.010216	13.820622
5. BC023105	188.44748	22.584827
6. Brix1	225.95549	29.568384
7. Cfb	6058.316	615.93933
8. Cxcr6	239.85176	28.186914
9. Cyp7b1	901.8936	115.928
10. Dhx58	219.885	12.213938
11. Eif2ak2	913.432	62.216557
12. Ereg	110.01636	553.9332
13. Fosb	26.025581	282.71332
14. Ggct	485.50192	56.232475
15. GM14085	186.08824	18.76144
16. GM16340	302.88657	20.148888
17. GM20559	2167.7356	179.40056
18. Gzma	164.543	164.543
19. Ido1	643.16724	61.43462
20. Ifi44	3003.7666	283.54318
21. Ighg2c	326.03513	15.449826
22. Iglv1	575.0526	32.583996
23. Kif1b	302.67603	37.680756
24. Lgals9	5191.8857	698.2904
25. Lipg	505.07483	40.0005
26. Lipt1	113.089005	15.379408

27. LOC1005039	165.35118	34.595467
28. Malat1	234.74217	28.271671
29. Ms4a4b	828.67255	67.354805
30. Myl1	176.43298	17.636652
31. Nmi	3526.5002	432.79007
32. Noxo1	410.87567	51.357624
33. Oas1a	2773.5667	280.92178
34. Oas3	514.51746	48.29396
35. Parp9	5948.464	766.62915
36. Pdcd1	112.29765	16.0185
37. Pla1a	165.39644	31.01755
38. Plac8	4131.4106	582.4446
39. Prg4	479.90997	58.986454
40. Psat1	182.66028	25.044243
41. Pus3	158.71689	21.603443
42. Sdc4	234.56674	10490.351
43. Sectm1b	773.29205	74.11949
44. Sephs2	470.59857	53.560066
45. Serpina3n	1496.6802	114.9884
46. Slc28a3	352.70645	28.834
47. Sp100	859.3947	113.937546
48. Tlr3	719.0728	76.4386
49. Tnfsf14	217.55724	23.653778
50. Tor3a	1371.1831	131.66748
51. Tpx2	197.23714	15.878368
52. Trim21	1148.3086	144.94237
53. Trim12a	113.0530	18.747263
54. Trim30d	1087.3447	37.673794
55. Trpm7	37.270153	201.78102
56. Zfp322a	234.74217	28.271671

**48h**	**48h C57 raw**	**48h ECRG4 KO raw**
1. Abca1	1599.0303	204.82614
2. Col1a1	10744.308	1135.1538
3. Ddx3y	9.198065	232.38129
4. Dhx58	166.48624	14.852264
5. Epb4.1l2	614.7918	90.11535
6. Grb10	159.92888	16.655273
7. Gusb	787.98663	115.4561
8. Igha	84.08041	619.4644
9. Il4ra	981.5553	115.34644
10. Mknk1	357.70297	32.03707
11. Myh1	13.157791	68.84086
12. Pla2g15	331.33627	36.38348

13. Prkd3	166.86238	14.21671
14. Rab31	393.77957	44.866833
15. Scpep1	3163.3958	410.16794
16. Sdc1	877.2603	128.46622
17. Tnfsf14	137.09158	16.917

Raw gene array values for interleukin- and interferon-related genes regulated more than 2-fold in the ECRG-null middle ear (Table 6)

**6h**	**6h C57 raw**	**6h ECRG4 KO raw**
1. IL11	100.25518	22.45559
2. Il12b	30.3439	41.72234
3. Il15	717.53534	96.82138
4. Il22	60.98509	16.369236
5. Il1r2	5146.824	1344.186
6. Il1rl1	702.6299	181.1449
7. Il1rn	1014.6925	126.61098
8. Il1f9	668.63654	75.954506
9. Il1rap	161.8764	27.603666
10. Il2ra	54.330772	14.761902
11. Il2rg	611.64984	208.8038
12. Il4ra	969.3748	312.30624
13. Il4i1	232.4207	36.949314
14. Il15ra	189.49892	63.780552
15. Il17ra	334.23386	90.12592
16. Il13ra1	1519.6163	336.83246
17. Ifnb1	35.31238	44.879707
18. Ifnar2	647.9719	250.43373
19. Ifngr2	239.0859	81.72313
20. Irf3	775.98193	303.97852
21. Irf5	274.27454	90.44762
22. Irf7	2869.1284	236.3659
23. Irf9	1339.3114	363.70193
