# Supplementary material for: The ECRG4 cleavage product augurin binds the endotoxin receptor and influences the innate immune response during otitis media
Source: Front Genet. 2022 Aug 26;13:932555. doi: 10.3389/fgene.2022.932555 (PMC9461705; doi:10.3389/fgene.2022.932555)
Supplement: Supplementary file 2 [file DataSheet1.PDF]

SUPPLEMENTARY TABLE 1. ECRG4 INTERACTING GENES EXPRESSION IN DIFFERENT ME CELL TYPES UPTO 2 DAYS POST NTHI INNOCULAITON. Expression above 0.1 threshold is in **Bold**. (EPIT: Epithelium, ENDO: Endothelium, STRO: Stromal, MONO: Monocytes, PMN: Polymorphonuclear leukocytes)

		EPIT	ENDO	STRO	MONO	PMN
Tlr4	0h	0.056	0.041	0.036	0.067	------
	6h	**1.148**	**0.936**	0.014	0.017	**0.249**
	1d	**0.236**	**0.167**	0.062	**0.315**	**0.401**
	2d	0.062	0.000	0.039	**0.119**	0.071
Cd14	0h	**0.471**	**0.474**	0.095	**2.807**	--------
	6h	**5.209**	**2.195**	**0.827**	**4.755**	**5.087**
	1d	**0.856**	**1.199**	**3.693**	**3.038**	**3.700**
	2d	**1.160**	**0.265**	**0.111**	**2.515**	**2.942**
MD2 (*Ly96*)	0h	0.082	**0.330**	**0.136**	**0.279**	-------
	6h	**0.298**	**0.103**	**0.427**	0.097	**0.168**
	1d	**0.176**	**0.25**8	0.046	**0.431**	**0.285**
	2d	**0.171**	0.021	0.012	**0.358**	**0.42**0
Olr1	0h	0.000	0.000	0.010	0.000	0.000
	6h	0.016	0.000	0.009	0.098	0.092
	1d	0.037	0.008	0.026	**0.169**	**0.282**
	2d	0.012	0.000	0.000	0.098	**0.149**
Scarf1	0h	0.004	**0.854**	0.013	0.008	-------
	6h	0.000	**0.421**	0.009	0.032	0.002
	1d	0.004	**0.478**	0.002	0.039	0.001
	2d	0.000	**0.495**	0.000	0.078	0.000
Cd36	0h	**0.523**	0.000	0.040	**0.275**	-------
	6h	**0.111**	0.037	0.004	**0.139**	0.000
	1d	0.014	0.020	0.009	**0.147**	0.001
	2d	0.007	0.022	0.000	**0.327**	0.000
Stab1	0h	0.019	**2.095**	0.031	**0.525**	-------
	6h	0.016	**0.685**	0.000	0.047	0.003
	1d	0.017	**1.560**	0.021	**0.913**	0.031
	2d	0.019	**0.802**	0.026	**1.282**	0.013

BOLD = >0.100
